# Editorial: Adaptation mechanisms of grass and forage plants to stressful environments

**DOI:** 10.3389/fpls.2023.1132198

**Published:** 2023-02-07

**Authors:** Jing Zhang, Mao-Feng Chai, Sergey Shabala, Ke-Hua Wang, Jin-Lin Zhang

**Affiliations:** ^1^ College of Agro-grassland Science, Nanjing Agricultural University, Nanjing, China; ^2^ Key Laboratory of National Forestry and Grassland Administration on Grassland Resources and Ecology in the Yellow River Delta, College of Grassland Science, Qingdao Agricultural University, Qingdao, China; ^3^ Tasmanian Institute of Agriculture, University of Tasmania, Hobart, TAS, Australia; ^4^ Department of Turfgrass Science and Engineering, College of Grassland Science and Technology, China Agricultural University, Beijing, China; ^5^ State Key Laboratory of Herbage Improvement and Grassland Agro-ecosystems; Key Laboratory of Grassland Livestock Industry Innovation, Ministry of Agriculture and Rural Affairs; College of Pastoral Agriculture Science and Technology, Lanzhou University, Lanzhou, China

**Keywords:** grass and forage plants, stress tolerance, natural metabolites, synthetic chemicals, host-microbe interactions, gene functional characterization, molecular breeding

Environments determine plant distribution and productivity in the world (Bailey-Serres et al., 2019). In nature, plants are constantly challenged by stressful environments, such as drought, heat, cold, nutrient deficiency, flooding, salinity and toxic heavy metals in the soil, insufficient or excessive light, and pathogens and pests, etc. (Zhang et al., 2022). These abiotic stresses limit the world-wide utilization of arable lands and negatively affect crop productivity (Bailey-Serres et al., 2019). There are growing concerns about continued global warming and increasing extreme weather events, which subsequently lead to frequent natural disasters and environmental problems for agricultural practice worldwide (Zandalinas et al., 2021; Verslues et al., 2023). Global population rose from 5 billion inhabitants in 1990 to more than 7.5 billion presently and will rise to 9.7 billion to 10 billion by 2050 (Gupta et al., 2020). The current pace of crop yield increase cannot meet the demand for future population (Hickey et al., 2019). Therefore, understanding the mechanisms on how plants adapt to stressful environments is critical for global ecological protection and food security.

Grasslands dominate terrestrial ecosystem on the earth, producing food, feed, fiber and fuel, and serving as weather amelioration, carbon sequestration, biodiversity enhancement, soil conservation, recreation, and the maintenance of the atmospheric composition (Bai and Cotrufo, 2022; Strömberg and Staver, 2022). Grass and forage plants serve multiple functions and benefits to humans and animals, such as beautifying landscapes, protecting the environments, improving human recreational activities, and providing feed for livestock and wild animals (Kopecký and Studer, 2014; Simeão et al., 2021) More importantly, grass and forage plants with rich biodiversity, especially including many wild species, have evolved multiple mechanisms to adapt to various stressful environments as described above at physiological, biochemical, molecular, cellular, and subcellular levels, compared to crop plants (Pardo and VanBuren, 2021; McSteen and Kellogg, 2022). Hence, it is urgently necessary to explore these mechanisms and the underlying strategies that will facilitate grass and forage plant breeding and crop plant breeding for improved stress tolerance. In this topic, recent research advances in adaptation mechanisms of grass and forage plants to stressful environments are presented in 40 research articles and one review article, contributed by 273 authors. The 40 research articles covered 23 plant genera and 30 species, 10 of which is about *Medicago*, including five in *Medicago sativa*, three in *Medicago truncatula*, one in *Medicago falcate* and another one in *Medicago ruthenica*, indicating that alfalfa as “the king of forage plants” still arouses the greatest concern of scientists in the field (**Figure 1**).

**Figure 1:**
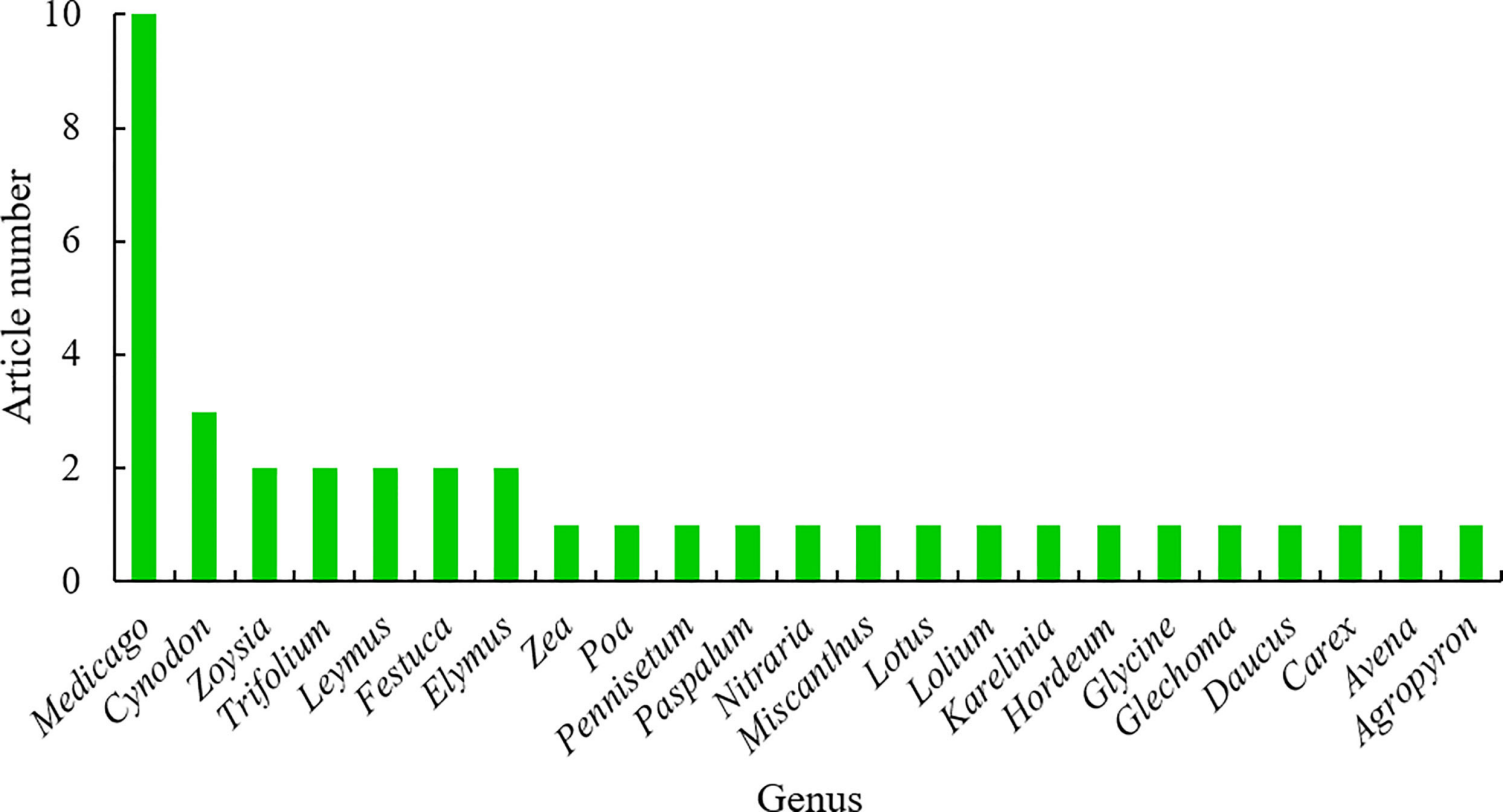
Genus distribution of 41 articles published in this topic.

## Functional characterization of genes relevant to stress tolerance

The processes of plant adaptation to stressful environments are controlled and regulated by multiple genes (Pardo and VanBuren, 2021; Zhang et al., 2022). Functional characterization of these genes is helpful to understand how grass and forage plants adapt to stressful environments and selected genes can be used for breeding grass, forage and crop plant cultivars with improved stress tolerance. Jiang et al. characterized a novel transcriptional regulator HbERF6 that regulates the HbCIPK2-coordinated pathway conferring salt tolerance in a halophytic grass *Hordeum brevisubulatum*. Zhou et al. constructed a high-density genetic map and localized grazing-tolerant QTLs in *Medicago falcata* L. Wang et al. identified *LjHDZ7* encoding a 40 HD-Zip transcription factor from *Lotus japonicas* and the overexpression of *LjHDZ7* increased plant salt tolerance. The overexpression of abscisic acid-insensitive gene (*ABI4*) from *Medicago truncatula* by Li et al. enhanced the content of endogenous ABA in plants and improved plant cold tolerance. Guan et al. found that *Zoysia japonica* ZjNOL promotes chlorophyll degradation and senescence and negatively affects the integrity and function of the photosystem.

## Regulations of stress tolerance by natural metabolites or synthetic chemicals

Numerous structurally different metabolites are produced in plants in response to various stressful environments (Kim et al., 2007; Kim et al., 2017). Li et al. found that caffeic acid O-methyltransferase gene *CrCOMT* from *Carex rigescens* conferred melatonin-mediated drought tolerance in plants. Differential responses of four white clover genotypes to salt stress associated with root growth, endogenous polyamines metabolism, and sodium/potassium accumulation and transport were identified by Li et al. Yu et al. identified 90 uridine diphosphate glycosyltransferase (UGT) members in ten evolutionary groups that are likely related to secondary metabolites in alfalfa (*Medicago sativa* L.). Li et al. found that the flexible response of a large number of genes and metabolites endows *Poa crymophila* with robust cold and drought tolerance. Yang et al. demonstrated that genotypic-specific reprogramming and crosstalk of various plant hormones are crucial for root growth and salt tolerance of bermudagrass (*Cynodon dactylon*). Lin et al. found that *Leymus chinensis* adapts to degraded soil environments by changing its metabolic pathways and root exudate components. Overexpression of *Pennisetum purpureum* CCoAOMT encoding caffeoyl-CoA O-methyltransferase by Song et al. contributes to lignin deposition and drought tolerance by promoting the accumulation of flavonoids in transgenic plants.

## Roles of host-microbe interactions in stress responses

Root-associated microbes can improve plant growth, and offer the potential to increase plant tolerance to stressful environments (Saijo and Loo, 2020; Vries et al., 2020). Mei et al. found that the planting of alfalfa can promote the proliferation of specific beneficial microbiota groups in the soil. Wang et al. demonstrated that *Bacillus amyloliquefaciens* GB03 augmented tall fescue growth by regulating phytohormone and nutrient homeostasis under nitrogen deficiency condition. Hou et al. found that *Bacillus atrophaeus* WZYH01 and *Planococcus soli* WZYH02 improved salt tolerance of maize (*Zea mays* L.). Zhang et al. demonstrated that inoculation of *Elymus nutans* with arbuscular mycorrhizal fungi *Funneliformis mosseae* improved the uptake of nutrients and induced the resistance to grasshopper attack. Pan et al. found that root exudates and rhizosphere soil bacterial relationships of *Nitraria tangutorum* are linked to k-strategist bacterial community under salt stress. Wei et al. demonstrated that salt-tolerant endophytic bacterium *Enterobacter ludwigii* B30 enhance bermudagrass growth under salt stress by modulating plant physiology and changing rhizosphere and root bacterial community.

## Omics-related studies in stress tolerance of grass and forage plants

Recent significant progress in omics techniques (transcriptomics, genomics, proteomics, and metabolomics) have helped to deeply understand the molecular insights into multiple stress tolerance of plants (Singhal et al., 2021). Salt tolerance in alfalfa is associated with regulation of ionic homeostasis, antioxidative enzymes and fatty acid metabolism at both transcriptional and physiological level (Li et al.). Transcriptomic profiling by Chen et al. showed the role of 24-epibrassinolide in alleviating salt stress damage in tall fescue (*Festuca arundinacea*). A transcriptome analysis by Dong et al. revealed the molecular regulatory mechanisms of leaf senescence in *Medicago truncatula* under alkaline stress. Another transcriptome analysis by Li et al. revealed the molecular response mechanism of high-resistant and low-resistant alfalfa varieties to *Verticillium alfalfa.* A combined analysis of the transcriptome and proteome by Ming et al. revealed the mechanisms underlying the enhanced salt tolerance by the protein disulfide isomerase gene (*ZmPDI*) in *Zoysia matrella* [L.] Merr. An integrated analysis of small RNAs, transcriptome and degradome sequencing by Fan et al. revealed the drought stress network in *Agropyron mongolicum*. A series integrated analyses in *Medicago truncatula* in response to salt stress by An et al. revealed multiple differentially expressed coding and non-coding RNAs, including mRNAs, lncRNAs, circRNAs, and miRNAs, and they identified multiple DEmRNA and ceRNA interaction pairs that function in many pathways of salt stress responses.

Overall, the articles collected on this Research Topic represent a substantial contribution to fill gaps in knowledge of the roles of complex signaling transduction pathways in grass and forage plants in response to various stressful environments. Moreover, the stress tolerance-related genes, beneficial natural metabolites, and root-associated microbes identified are valuable resources not only for grass and forage plants, but also for other crops. Jiuxin and Liebao reviewed the research progress of turfgrass resistance breeding, analyzed the bottlenecks of turfgrass resistance breeding, and put forward the strategies to cope with the bottlenecks, which will be useful to guide turfgrass breeding for stress tolerance.

## Author contributions

J-LZ and JZ prepared the draft. JZ, M-FC, SG, K-HW and J-LZ revised the manuscript. All authors approved the final version of the manuscript and approved it for publication.

## References

[B1] Bai Y. Cotrufo M. F. (2022). Grassland soil carbon sequestration: Current understanding, challenges, and solutions. Science 377, 603–608. doi: 10.1126/science.abo2380 35926033

[B2] Bailey-Serres J. Parker J. E. Ainsworth E. A. Oldroyd G. E. D. Schroeder J. I. (2019). Genetic strategies for improving crop yields. Nature 575, 109–118. doi: 10.1038/s41586-019-1679-0 31695205 PMC7024682

[B3] Gupta A. Rico-Medina A. Cao-Delgado A. I. (2020). The physiology of plant responses to drought. Science 368, 266–269. doi: 10.1126/science.aaz7614 32299946

[B4] Hickey L. T. Hafeez A. N. Robinson H. Jackson S. A. Leal-Bertioli S. C. M. Tester M.. (2019). Breeding crops to feed 10 billion. Nat. Biotechnol. 37, 744–754. doi: 10.1038/s41587-019-0152-9 31209375

[B5] Kim J. K. Bamba T. Harada K. Fukusaki E. Kobayashi A. (2007). Time-course metabolic profiling in *Arabidopsis thaliana* cell cultures after salt stress treatment. J. Exp. Bot. 58, 415–424. doi: 10.1093/jxb/erl216 17118972

[B6] Kim J. M. To T. K. Matsui A. Tanoi K. Kobayashi N. I. Matsuda F.. (2017). Acetate-mediated novel survival strategy against drought in plants. Nat. Plants 3, 17097. doi: 10.1038/nplants.2017.97 28650429

[B7] Kopecký D. Studer B. (2014). Emerging technologies advancing forage and turf grass genomics. Biotechnol. Adv. 32, 190–199. doi: 10.1016/j.biotechadv.2013.11.010 24309540

[B8] McSteen P. Kellogg E. A. (2022). Molecular, cellular, and developmental foundations of grass diversity. Science 377, 599–602. doi: 10.1126/science.abo5035 35926032

[B9] Pardo J. VanBuren R. (2021). Evolutionary innovations driving abiotic stress tolerance in C4 grasses and cereals. Plant Cell 33, 3391–3401. doi: 10.1093/plcell/koab205 34387354 PMC8566246

[B10] Saijo Y. Loo E. P. (2020). Plant immunity in signal integration between biotic and abiotic stress responses. New Phytol. 225, 87–104. doi: 10.1111/nph.15989 31209880

[B11] Simeão R. M. Resende M. D. V. Alves R. S. Pessoa-Filho M. Azevedo A. L. S. Jones C. S.. (2021). Genomic selection in tropical forage grasses: current status and future applications. Front. Plant Sci. 12. doi: 10.3389/fpls.2021.665195 PMC8120112 33995461

[B12] Singhal R. K. Saha D. Skalicky M. Mishra U. N. Chauhan J. Behera L. P.. (2021). Crucial cell signaling compounds crosstalk and integrative multi-omics techniques for salinity stress tolerance in plants. Front. Plant Sci. 12. doi: 10.3389/fpls.2021.670369 PMC8414894 34484254

[B13] Strömberg C. A. E. Staver A. C. (2022). The history and challenge of grassy biomes. Science 377, 592–593. doi: 10.1126/science.add1347 35926015

[B14] Verslues P. E. Bailey-Serres J. Brodersen C. Buckley T. N. Conti L. Christmann A.. (2023). Burning questions for a warming and changing world: 15 unknowns in plant abiotic stress. Plant Cell 35, 67–108. doi: 10.1093/plcell/koac263 PMC9806664 36018271

[B15] Vries F. Griffiths R. I. Knight C. G. Nicolitch O. Williams A. (2020). Harnessing rhizosphere microbiomes for drought-resilient crop production. Science 368, 270–274. doi: 10.1126/science.aaz5192 32299947

[B16] Zandalinas S. I. Fritschi F. B. Mittler R. (2021). Global warming, climate change, and environmental pollution: Recipe for a multifactorial stress combination disaster. Trends Plant Sci. 26, 588–599. doi: 10.1016/j.tplants.2021.02.011 33745784

[B17] Zhang H. Zhu J. Gong Z. Zhu J. K. (2022). Abiotic stress responses in plants. Nat. Rev. Genet. 23, 104–119. doi: 10.1038/s41576-021-00413-0 34561623

